# Relationship between the transmural extent of high resolution parametric (T1- and T2) mapping and the ischemic area at risk defined by pathology

**DOI:** 10.1186/1532-429X-18-S1-O44

**Published:** 2016-01-27

**Authors:** Dave Wendell, Han W Kim, Elizabeth Jenista, Wolfgang G Rehwald, Michele Parker, Enn-Ling Chen, Pairoj Chattranukulchai, Robert Judd, Raymond Kim

**Affiliations:** 1 grid.26009.3d0000000419367961Medicine/Cardiology DCMRC, Duke University, Durham, NC USA; 2 Siemens Healthcare, Chicago, IL USA

## Background

Delineating the area-at-risk (AAR) is crucial in determining the amount of salvaged myocardium following an acute myocardial infarction (MI). Myocardial parametric mapping (native T1- and T2-mapping) has been promoted as an excellent method to delineate the AAR, and image reproducibility appears to be superior to that for black-blood TSE imaging [1]. However, robust validation of these techniques with a direct comparison of the transmural shape of the AAR by pathology with the transmural shape of the abnormal regions in parametric maps has not been performed.

## Methods

We used a canine model of acute MI with variable coronary occlusion times (40-90 min) to create a range of infarct sizes and transmurality (n = 12). We obtained high spatial resolution T1 and T2 maps (0.75 × 0.75 × 6 mm) to reduce the magnitude of partial volume effects in altering T1 and T2 measurements. CMR was performed 2-5 days following MI at 3T (Siemens Verio). The AAR and MI were delineated by pathology using microspheres and TTC staining, respectively. Abnormal regions on MRI were compared to pathology by quantitative measurement of the transmural-extent of the abnormality (abnormal area/sector area). T1 and T2 values were measured within the infarcted and salvaged portions of the AAR and in remote myocardium. CMR was also performed in five patients with acute MI for which imaging and analysis were performed similar to that for canines.

## Results

**Figure 1:**
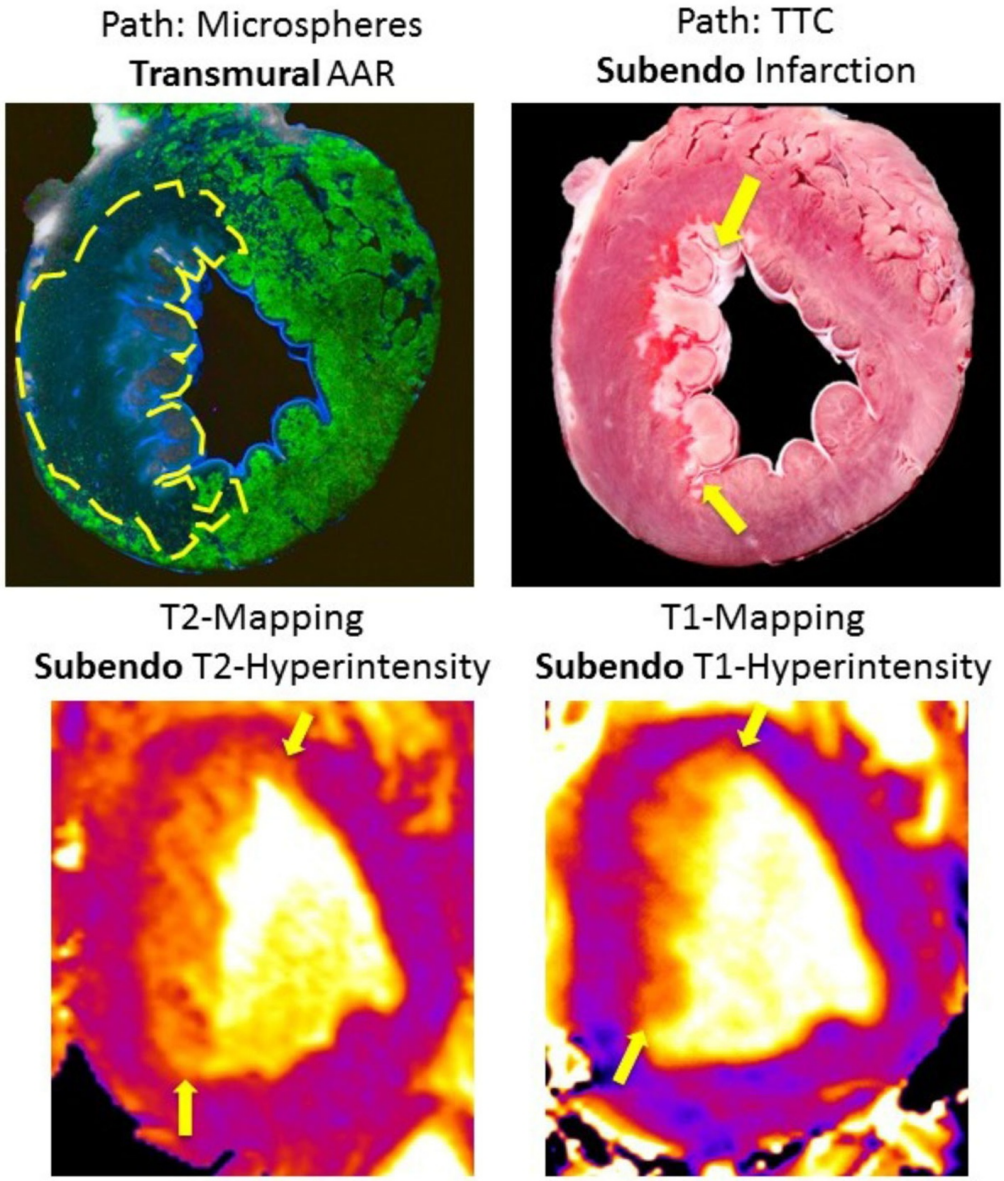
**Microsphere image (top, left) demonstrating fully transmural AAR, with subendocardial infarction shown on TTC (top, right)**. In vivo T1 and T2 abnormalities are also limited to the subendocardium (bottom)

**Figure 2:**
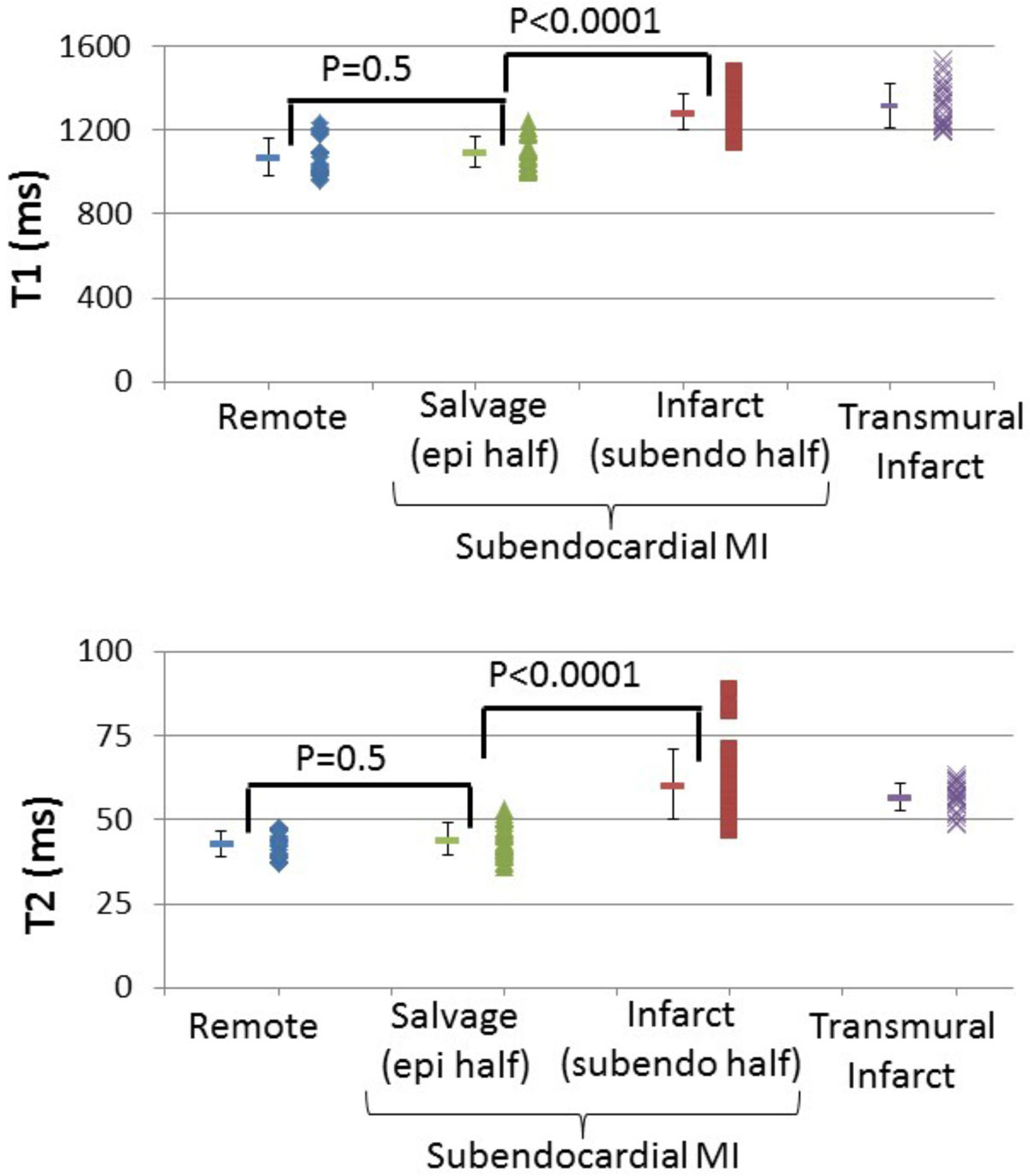
**T1 and T2 measurements showing the epicardial half of the AAR is no different than remote myocardium**.

Analysis of the 60 matched slices in canines demonstrated there was no relationship between the transmural extent of elevated T1 and T2 regions on MRI and that of the AAR delineated by microspheres (T1: r = -0.28, p = 0.07, T2: r = -0.13, p = 0.44). Instead, there was a strong correlation with that of infarction by TTC (T1: r = 0.90, p < 0.0001, T2: r = 0.86, p < 0.0001). Of 32 slices with subendocardial MI (< 50% transmural) confirmed by pathology, 30 (94%) and 30 had T1 and T2 abnormalities that were limited to the subendocardium, respectively (Fig. 1). Of these, the T1 and T2 values in the epicardial half of the AAR (i.e. salvaged myocardium) were no different than that of remote (Fig 2). Similarly in patients, a strong correlation existed between the transmural extent of T1 and T2 abnormalities and the transmural extent of infarction by DE-MRI (T1: r = 0.970, T2: r = 0.973), and in those patients with subendocardial infarction, T1 and T2 abnormalities were limited to the subendocardium.

## Conclusions

With high resolution T1 and T2 mapping, no relationship was seen between the transmural extent of T1 and T2 abnormalities and that of the AAR by pathology. Instead there was a strong correlation with the transmural extent of infarction. The use of native T1 and T2 mapping to delineate the AAR is problematic.

## References

[1] Bulluck (2015). JCMR.

